# Alterations and Correlations of Gut Microbiota and Fecal Metabolome Characteristics in Experimental Periodontitis Rats

**DOI:** 10.3389/fmicb.2022.865191

**Published:** 2022-04-14

**Authors:** Lan Wu, Jie Han, Jia-Yan Nie, Tong Deng, Cheng Li, Cheng Fang, Wen-Zhong Xie, Shuang-Ying Wang, Xian-Tao Zeng

**Affiliations:** ^1^Center for Evidence-Based and Translational Medicine, Zhongnan Hospital of Wuhan University, Wuhan, China; ^2^Department of Stomatology, Zhongnan Hospital of Wuhan University, Wuhan, China; ^3^Department of Gastroenterology, Zhongnan Hospital of Wuhan University, Wuhan, China; ^4^Department of Stomatology, Kaifeng University Health Science Center, Kaifeng, China

**Keywords:** periodontitis, fecal metabolites, KEGG functional pathway, periodontal disease, gut microbiota, multi-omics analysis

## Abstract

**Objectives:**

Periodontitis affects the progression of many diseases, while its detailed mechanism remains unclear. This study hopes to provide new ideas for exploring its mechanism by analyzing the gut microbiota and fecal metabolic characteristics of experimental periodontitis rats.

**Methods:**

A total of 10 rats were randomly divided into ligature-induced experimental periodontitis (EP) group and healthy control group. After 4 weeks of the experiment, the feces of all rats were collected for sequencing through 16S ribosomal DNA (rDNA) sequencing technology and liquid chromatography–mass spectrometry (LC–MS).

**Results:**

16S rDNA sequencing results showed that the β-diversity of gut microbiota was significantly different between the EP and control group, and the levels of dominant genera were different. Compared with the control group, *Ruminococcus*, *Escherichia*, and *Roseburia* were significantly enriched in EP, and *Coprococcus*, *Turicibacter*, *Lachnospira* were significantly decreased. Correlation analysis showed that *Roseburia* exhibited the highest correlation within the genus. Of 3,488 qualitative metabolites, 164 metabolites were upregulated and 362 metabolites were downregulated in EP. Enrichment analysis showed that periodontitis significantly changed 45 positive/negative ion metabolic pathways. Five KEGG pathways, protein digestion and absorption, tyrosine metabolism, glycolysis/gluconeogenesis, niacin and nicotinamide metabolism, and oxidative phosphorylation, are enriched in both the microbiome and metabolome. Correlation analysis showed that the genera with significant differences in periodontitis were usually significantly correlated with more metabolites, such as *Roseburia*, *Lachnospira*, *Escherichia*, *Turicibacter*, and *Ruminococcus*. The genera with the same changing trend tended to have a similar correlation with some certain metabolites. In addition, vitamin D2 and protoporphyrin IX have the most significant correlations with microorganisms.

**Conclusion:**

Our study reveals that periodontitis alters gut microbiota and fecal metabolites. The correlation analysis of microbiota and metabolome provides a deeper understanding of periodontitis, and also provides a direction for the study of periodontitis affecting other diseases.

## Introduction

Periodontitis is an inflammatory disease caused by pathogenic microorganisms in the oral cavity, and the culprit is the dental plaque that grows on the surface of the teeth (Darveau, 2010). Patients with severe periodontitis may swallow 10^12^–10^13^ bacteria in saliva every day (Sender et al., 2016). A growing number of studies have shown that periodontitis is involved in the progression of various diseases, including metabolic syndrome, type 2 diabetes mellitus (T2D), benign prostatic hyperplasia, chronic kidney disease (CKD), coronary heart disease, and cancer (Tonetti and Van Dyke, 2013; Genco and Sanz, 2020; Fang et al., 2021b; Yuan et al., 2021). Studies suggest that its possible mechanisms include distant spread of periodontopathogens, ectopic infection, distal end migration of oral inflammatory–activated Th17 cells, and metabolic and immune disorders (Kitamoto et al., 2020; Fang et al., 2021b; Hajishengallis and Chavakis, 2021), but the specific mechanism is still not clear. Gut microbiota disorder is increasingly considered as the pathogenesis of many diseases. More and more studies have reported that periodontal pathogens can affect gut microbiota dysbiosis through ectopic colonization, pathological factors, or virulence factors (Yamazaki et al., 2020; Hajishengallis and Chavakis, 2021). Gut microbiota disturbance may be one of the mechanisms of periodontitis affecting distal diseases (Huang et al., 2020), but the detailed mechanism remains to be fully elucidated.

Metabolites are usually measured in clinical medicine as biomarkers for diagnosis, prognosis, or therapeutic response (Wishart, 2019), providing a new entry point for exploring the pathogenesis of the disease. Li et al. (2020) suggest that gut microbial alterations caused by periodontitis can lead to systemic inflammation and metabolic dysfunction, which will accelerate the progression of T2DM. Sakanaka et al. (2021) explored the potential link between periodontitis and cardiometabolic diseases by studying metabolites. Previous studies have pointed out that the use of metabolomics to study the relationship between periodontitis and obesity gets significantly more outputs than the pure oral microbiome (Chen et al., 2021). At present, 16S rDNA sequencing technology is mostly used in the analysis of the gut microbiome, but this technology has the limitation of lacking quantitative functional annotation. Fecal metabolomics can better interpret the metabolic interactions among host, diet, and gut microbiota, and provide microbiome function data to supplement the deficiency of sequencing (Marcobal et al., 2013). The combination of metabolomics and microbiome is increasingly used to study the pathogenesis of diseases (Zierer et al., 2018; Shi et al., 2020; Na et al., 2021). Understanding the mechanism of the link between periodontitis and comorbidities is the focus of future translational research, and combined microbial and metabolic multi-omics analysis may provide a better understanding of the underlying mechanisms by which periodontitis affects other diseases.

Liquid chromatography–mass spectrometry (LC–MS) is a widely used metabolomics research technique with high resolution, high throughput, high sensitivity, and high specificity. In this study, we constructed an experimental periodontitis model and performed multi-omics analysis of all rats’ feces, that is, a combination of microbiome analysis using 16S rDNA V3-V4 region sequencing and metabonomic analysis using LC–MS. We first analyzed the impact of periodontitis on the gut microbiota and fecal metabolites, and then analyze their correlations and participating pathways in detail. The combination of microbiome and metabolomics can effectively provide sufficient and accurate data to reveal the influence of periodontitis. It could provide ideas for studying the potential mechanism of periodontitis affecting other diseases.

## Materials and Methods

### Experimental Periodontitis Rat Model and Sample Collection

Ten 7-week-old male Sprague–Dawley (SD) rats (purchased from Beijing Vital River Laboratory Animal Technology Co. Ltd., Beijing, China) were randomly divided into two groups (*n* = 5 each group). We conducted the adaptability of feeding for 1 week before the experiment. One group did not take additional treatments (Control), the other group was ligated with sterile nylon thread around the cervical of bilateral maxillary first and second molars to induce experimental periodontitis (EP). The construction of the EP model and animal experiments were consistent with our previous study (Fang et al., 2021a). After 4 weeks of the experiment, the feces of each rat were collected using sterile cryopreservation tubes with a unique label, and frozen at −80°C within 3 h after sampling (Sato et al., 2021). Bilateral maxillary alveolar bone–contained molars were harvested and fixed with 4% paraformaldehyde. Rats were cared for in accordance with China’s “Animal Care and Use Guide.” This study conformed to the ARRIVE guidelines and was approved by the Animal Ethics Committee of Wuhan University (IACUC 2018119). Details are further described in the Supplementary Material.

### Microcomputed Tomographic and Histopathological Analyses of Alveolar Bone

Microcomputed tomographic (Skyscan 1176, Bruker, Kontich, Belgium) was used to evaluate the alveolar bone loss in each group. Three-dimensional (3D) reconstruction was performed by the software NReCon (Bruker, Kontich, Belgium) after scanning. And then the 3D images were obtained by the software CTvox (Bruker, Kontich, Belgium). The periodontal attachment loss was measured by DataViewer 1.5.2.4 (Bruker, Kontich, Belgium) for the maxillary second molar of four sites (the proximal buccal, proximal palatal, distal buccal, and distal palatal), and then the average value of periodontal attachment loss was calculated for statistical analysis.

The residual side of the maxillae was decalcified with 10% ethylenediaminetetraacetic acid (EDTA) disodium salt for 1 month and then embedded in paraffin. And then, from the occlusal surface of the crown to the alveolar bone, 4 μm sections were prepared along a plane parallel to the long axis of the tooth. The sections were standardly stained with hematoxylin and eosin (H and E), and images were captured with a light microscope (Leica DFC295, Wetzlar, Germany).

### DNA Extraction

According to the manufacturer’s instructions, we used MagPure Stool DNA KF kit B (Magen, Guangdong, China) to extract microbial genomic DNA from feces. The quantity and quality of DNA were checked by Qubit dsDNA BR Assay kit (Invitrogen, Carlsbad, CA, United States) with a Qubit Fluorometer and 1% agarose gel, respectively.

### 16S rDNA Microbiome Sequencing

The 16S rDNA amplicon (V3–V4 region) sequencing was performed on the Illumina HiSeq 2500 (BGI, Shenzhen, China) platform. The primers were all processed with Illumina standard method, and the sequence used was 341F (5′-ACTCCTACGGGAGGCAGCAG-3′) and 806R (5′-GGACTACHVGGGTWTCTAAT-3′). A 30 ng of qualified genomic DNA samples and the corresponding fusion primers were taken to configure the PCR reaction system. The PCR products were purified and eluted with AmpureXP beads to construct the library. We obtained 2 × 300 bp paired-end reads and the raw data have been deposited under NCBI BioProject accession numbers PRJNA778630 (EP) and PRJNA762590 (Control).

### Untargeted Metabolomics

Waters 2D UPLC (Waters, Milford, CT, United States) tandem Q Exactive HF high-resolution mass spectrometer (Thermo Fisher Scientific, Waltham, MA, United States) was used to separate and detect metabolites (see Supplementary Material for mobile phase and chromatographic separation). Samples (5 μl) were detected on a BEH C18 column (1.7 μm 2.1*100 mm, Waters, United States) at 45°C at a flow rate of 0.35 ml/min. Q Exactive HF mass spectrometer (Thermo Fisher Scientific, United States) was used for primary and secondary mass spectrometry data acquisition. Primary and secondary resolutions are 120,000 and 30,000, respectively. The samples were analyzed in positive ion (Spray voltage was 3.8 kV) and negative ion (Spray voltage was 3.2 kV) modes. The mass scanning range was 70–1050 *m/z*, and the ion transfer tube temperature was 320°C. The flow rates of sheath gas and auxiliary gas were set to 40 L/min and 10 L/min, respectively.

### Bioinformatic Analysis and Visualization

The total number of raw reads obtained by sequencing was 1,411,750, filtered to remove adaptors, low-quality, and ambiguous bases, and finally, 1,361,514 clean reads were obtained, with an average of 68,075.7*2 reads per sample. The detailed analysis steps of OTU clustering, chimera removal, α-diversity, β-diversity, difference comparison, and functional prediction are shown in the Supplementary Material.

Compound Discoverer 3.1 (Thermo Fisher Scientific, United States) was used to process the mass spectrum raw data and identify metabolites. R software package metaX (Wen et al., 2017) was used for data preprocessing, statistical analysis, metabolite classification annotation, and functional annotation. Principal component analysis (PCA) was used to analyze the overall distribution and stability between and within groups. Then, Partial Least-Squares Method–Discriminant Analysis (PLS–DA) was used to analyze the differences between groups and calculate Variable importance for the projection (VIP) of metabolites. To ensure model quality, sevenfold cross-validation was performed during modeling and validated by 200 response permutation testing. The obtained VIP values, combined with Fold Change (FC) obtained by univariate analysis, and *p* obtained by Student’s *t*-test were used to identify differential metabolites. Kyoto Encyclopedia of Genes and Genomes (KEGG) is a database for analyzing higher-order functions based on genomic information. KEGG pathway enrichment analysis was obtained by using hypergeometric tests to compare the significant difference metabolites with all identified metabolites as a background.

Spearman’s rank correlation coefficient was used to analyze the correlation between microbiota and metabolites, and visualization was made in the form of a heat map.

### Statistical Analyses

Data were evaluated using the Wilcoxon rank-sum test for two-group comparisons using the Wilcox-test package in R (v3.4.1). Correlations were identified by Spearman’s rank correlation coefficient (significance thresholds were *p* < 0.05) using the corrplot package in R (v3.4.1). GraphPad Prism V.8.0 (GraphPad Software Inc., San Diego, CA, United States) and R are used for various analyses and chart preparation. *p < 0.05, ^**^p < 0.01 and ^***^p < 0.001 were considered significant.

## Results

### Establishment of Experimental Periodontitis and Detection of Gut Microbiota

**FIGURE 1:**
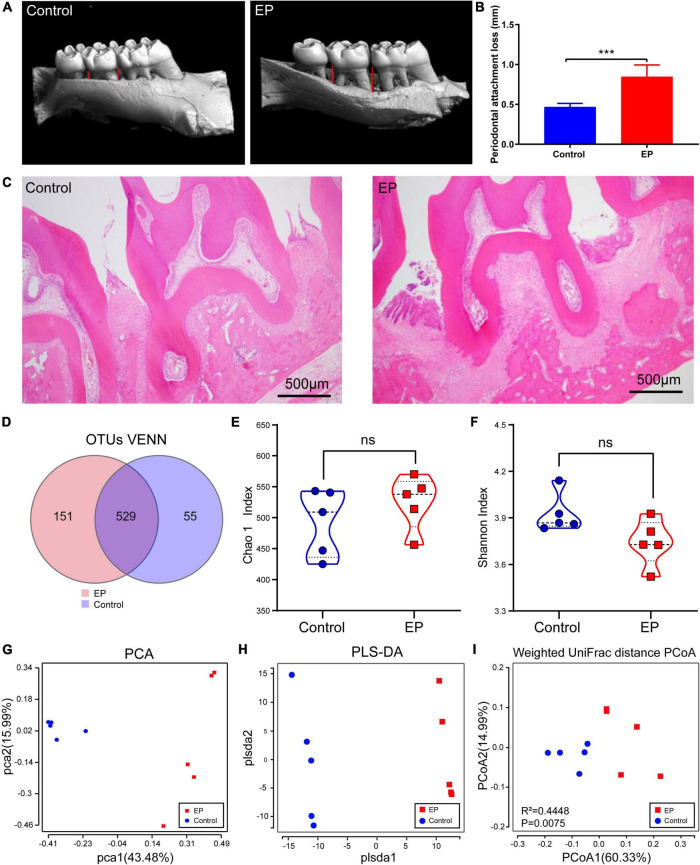
Detection of periodontal tissue in rats and diversity analysis of gut microbiota. **(A)** Representative 3D reconstruction images of alveolar bone by micro-CT. **(B)** Histogram of statistical analysis of periodontal attachment. **(C)** Representative figures from H and E staining for the second molar. **(D)** Shared and unique OTUs in the gut microbiome in EP and control group. Chao 1 index **(E)** and Shannon index **(F)** of the two groups. Principal component analysis (PCA) analysis **(G)**, partial least-squares method–discriminant analysis (PLS–DA) **(H)**, and Principal Coordinate Analysis (PCoA) based on Weighted Unifrac distance matrix **(I)** of EP and Control group. EP, experimental periodontitis group; Control, healthy control group. ***p < 0.001; ns, no significance.

Microcomputed tomographic was used to obtain 3D reconstruction images of alveolar bone, and the maxillary alveolar bone morphology was displayed (Figure 1A). Compared with the control group, obvious absorption of alveolar bone and exposure of roots were observed in the EP group. In addition, statistical analysis showed that the periodontal attachment loss in the EP group was significantly higher than that in the control group (Figure 1B). And the results of the H and E staining section (Figure 1C) focused on the periodontal tissue of the second molar. The images from EP groups presented gingival epithelium erosion with infiltrated inflammatory cell, collagen fiber breakdown, and resorption of alveolar bone. While no obvious pathological changes were observed in the control group. Taken together, these results confirmed that the experimental periodontitis was successfully induced.

The rarefaction curve of gut microbial 16S rDNA sequencing shows that the sample’s coverage is almost approached 100% (>99.8%), indicating that the sequencing depth of this study is sufficient to reflect most bacterial characteristics of the samples (Supplementary Figure 1A). A total of 735 OTUs have been detected by clustering the clean reads with a threshold of 97% (Figure 1D). EP and control groups had 529 OTUs in common, 151 OTUs were unique in the EP group and 55 OTUs were unique in the control group. α-Diversity analysis showed that compared with the control group, slightly more OTUs (Supplementary Figure 1B), decreased abundance (Chao1), and increased diversity (Shannon) were observed in the EP group, but the differences were not statistically significant (Wilcoxon signed-rank test, *p* > 0.05) (Figures 1E,F).

β-Diversity considers the differences in the composition of bacterial communities in different groups. PCA and PLS–DA analysis scatter plot showed that gut microbiota in the EP group was significantly different from that in the control group (Figures 1G–I). In the principal coordinate analysis (PCoA) of the weighted UniFrac distance (Figure 1I; *p* = 0.0075) and unweighted UniFrac distance (Supplementary Figure 1C; *p* = 0.0089), both the EP and control groups were well-separated, suggesting that periodontitis alters the structure of gut microbiota.

### Periodontitis Alters Gut Microbiota Composition

**FIGURE 2:**
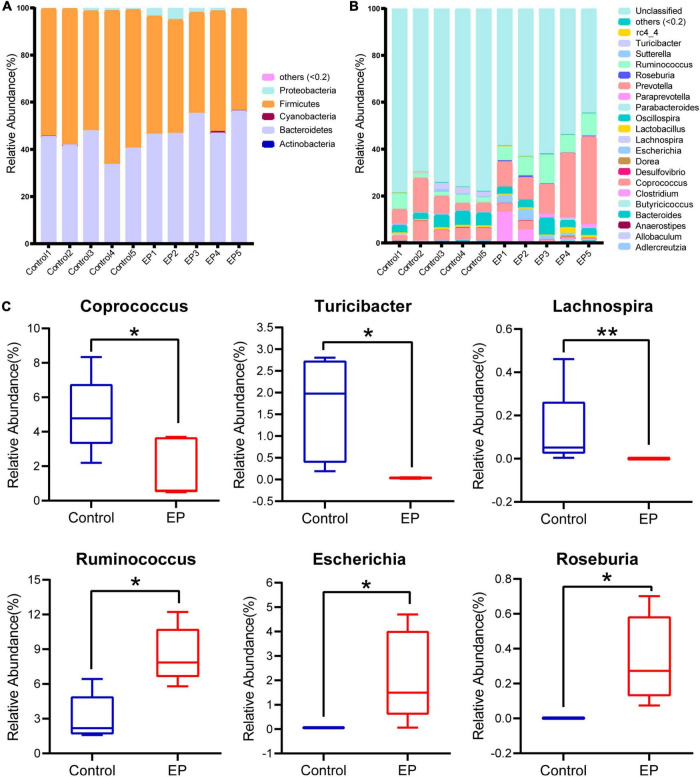
Differences in gut microbiota profile between EP and Control. Relative abundance of fecal microbiota composition at the level of phylum **(A)** and genus **(B)** of fecal samples in EP and Control. **(C)** Significantly different gut microbiota at genus level in EP compared with Control. *p < 0.05 and **p < 0.01. EP, experimental periodontitis group; Control, healthy control group.

To further understand the differences of microbial communities between EP and control group, we classified OTUs by comparing them with the Greengene database, and obtained the species abundance of each sample at phylum and genus levels. Species with abundance below 0.2% in all samples were merged as others, and the species without taxonomic-level annotations were merged as unclassified. At the phylum level, *Actinobacteria*, *Bacteroidetes*, *Cyanobacteria*, *Firmicutes*, and *Proteobacteria* account for more than 99% of the bacteria (Figure 2A). Among them, *Firmicutes* and *Bacteroidetes* were dominant. Compared with the control group, *Firmicutes* was significantly decreased in the EP group (57.3% vs. 47.13%, *p* < 0.05; Supplementary Figure 2).

A total of 21 genera had a relative abundance of more than 0.2% in at least one sample. The five dominant genera in the EP group were *Prevotella* (19.60%), *Ruminococcus* (8.51%), *Oscillospira* (3.93%), *Clostridium* (3.87%), and *Escherichia* (2.14%), but the ranking of dominant bacteria in the control group was different (Figure 2B). Compared to the control group, there were six significantly different genera in the EP group (*p* < 0.05; Figure 2C), in which three genera (*Coprococcus*, *Turicibacter*, *Lachnospira*) decreased, and three genera (*Ruminococcus*, *Escherichia*, *Roseburia*) increased. The LEfSe results further confirmed the difference in bacteria between EP and the control group (Supplementary Figure 3). These results suggest that gut microbiota is altered during the development of periodontitis.

**FIGURE 3:**
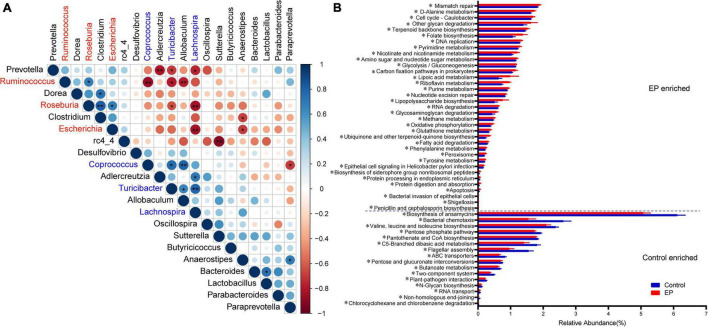
Relationships and predicted functions of gut microbiota. **(A)** Spearman’s rank correlation matrix of bacterial genera with >0.2% abundance in at least one sample. Circle size increases with absolute correlation and circle color denotes the nature of the correlation. On the scale, 1 indicates a complete positive correlation (dark blue) and −1 indicates a complete negative correlation (dark red) between the two microbial genera. Significant correlation between genera (*p < 0.05 and **p < 0.01). **(B)** Differentially enriched KEGG functions pathways (level 3) between EP and Control by PICRUST2 analysis. EP, experimental periodontitis group; Control, healthy control group.

The ecological connection and interaction among different genera were further investigated through Spearman’s correlation analysis. As shown in Figure 3A, *Roseburia*, enriched in the EP group, was associated with the highest number of microorganisms. It was significantly positively correlated with *Clostridium*, *Escherichia*, and *Ruminococcus*, and was co-excluded with *Turicibacter* and *Lachnospira*. *Turicibacter*, which was enriched in the control group, showed the strongest correlation. It was significantly positively correlated with *Prevotella*, *Ruminococcus*, and *Roseburia*, and significantly negatively correlated with *Coprococcus*, *Allobaculum*, and *Lachnospira*. The above results suggest that periodontitis induces tremendous changes and complex connections in the gut microbiota.

### Effect of Periodontitis on Functional Pathways

PICRUSt2 is a species-based bacterial community function prediction tool (Douglas et al., 2020), and we used it to predict the function of gut microbes. Figure 3B and Supplementary Figure 4 depict the significantly different functions of the two groups and focus on differences in metabolic functions. There were several pathways significantly enriched in the experimental periodontitis, including the metabolism of cofactors and vitamins (ubiquinone and another terpenoid–quinone biosynthesis, riboflavin metabolism, lipoic acid metabolism, nicotinate and nicotinamide metabolism, folate biosynthesis) and energy metabolism (oxidative phosphorylation, methane metabolism, carbon fixation pathways in prokaryotes). However, carbohydrate metabolism (butanoate metabolism, pentose, and glucuronate interconversions, C5-branched dibasic acid metabolism, pentose phosphate pathway) was significantly reduced in the experimental periodontitis. We found that periodontitis significantly affected the KEGG functional pathways by affecting the gut microbiota.

### Periodontitis Disrupts the Fecal Metabolome

**FIGURE 4:**
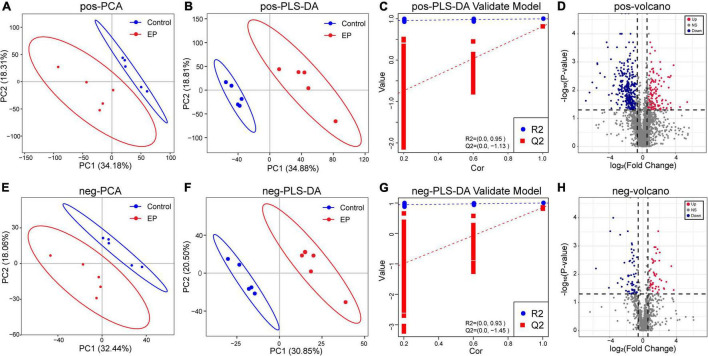
Differences of metabolic profiles between EP and Control groups based on LC–MS technology. Principal component analysis (PCA) analysis of positive ions **(A)** and negative ions **(E)**. Partial least-squares method–discriminant analysis (PLS–DA) analysis of positive ions **(B)** and negative ions **(F)**. The response permutation testing of PLS–DA for positive ions **(C)** and negative ions **(G)**. The volcano chart shows the difference in positive ion metabolites **(D)** and the difference in negative ion metabolites **(H)** between EP and Control groups. The red point means upregulation, blue point means downregulation. EP, experimental periodontitis group; Control, healthy control group; pos, positive ions; neg, negative ions.

We further used LC–MS to analyze metabolites in feces to assess whether periodontitis disrupts the metabolome of rats. The base peak chromatogram of LC–MS experiments in positive ion modes and negative ion modes are shown in Supplementary Figures 5, 6. A total of 2,632 qualitative positive-ion metabolites and 856 qualitative negative-ion metabolites were identified. PCA analysis was used to reveal global metabolic changes in EP and control rats, and the results showed that the two groups were distinguishable but not very significant (Figures 4A,E). To capture unique metabolic model and to maximize the distinction between the EP and control group metabolites, we applied the PLS–DA model. Results showed good modeling and prediction capabilities, and the metabolite clusters of EP and control group were well-separated (Figures 4B,C,F,G). VIP value, FC, and two-tailed Student’s *t*-test were used to distinguish differential metabolites, and the volcano plot of metabolites is shown in Figures 4D,H. Compared with the control group, 122 positive-ion and 42 negative-ion metabolites were significantly increased in the EP group (*p* < 0.05, VIP > 1, and FC ≥ 1.5), and 301 positive-ion and 61 negative-ion metabolites were significantly decreased in the EP group (*p* < 0.05, VIP > 1, and FC ≤ 0.67). KEGG database was used to annotate all the differential metabolites, among which 27 positive-ion and 15 negative-ion metabolites participated in the signaling pathway and were taken as key metabolites (Supplementary Table 1).

### Kyoto Encyclopedia of Genes and Genomes Pathways Analysis and Enrichment

**FIGURE 5:**
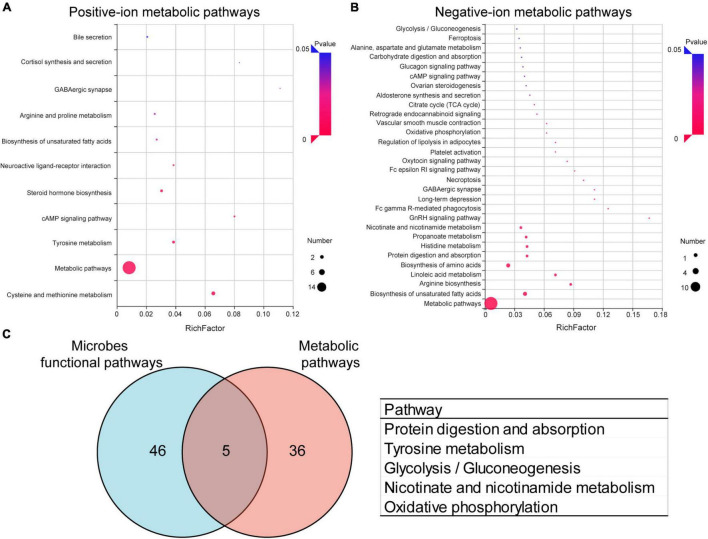
Effects of periodontitis on gut microbe and fecal metabolism. Enrichment of positive-ion metabolic pathways **(A)** and negative-ion metabolic pathways **(B)** in EP vs. Control. **(C)** Venn diagram of differential metabolic enrichment pathway and the predictive differential functional pathway of gut microbes, the table shows the KEGG pathway in common. EP, experimental periodontitis group; Control, healthy control group; pos, positive ions; neg, negative ions.

To further analyze the effects of periodontitis on metabolism, KEGG pathway annotation and enrichment analysis were performed on metabolites in both the groups. The metabolic annotation pathways and the number of corresponding upregulated and downregulated metabolites are shown in Supplementary Figures 7, 8. The results showed that these differential metabolites were mainly involved in metabolism, focusing on amino acid metabolism, lipid metabolism, and carbohydrate metabolism. The enrichment results showed that 11 positive-ion metabolic pathways (Figure 5A) and 34 negative-ion metabolic pathways (Figure 5B) were significantly enriched (Supplementary Table 2). The main positive-ion metabolic pathways involved were cysteine and methionine metabolism, tyrosine metabolism, and cAMP signaling pathway. The main negative-ion metabolic pathways involved were biosynthesis of unsaturated fatty acids, arginine biosynthesis, and biosynthesis of amino acids. The hub pathways of microbiota and metabolome were protein digestion and absorption, tyrosine metabolism, glycolysis/gluconeogenesis, nicotinate and nicotinamide metabolism, and oxidative phosphorylation, as shown in the Venn diagrams (Figure 5C).

### Correlation Between Gut Microbiota and Fecal Metabolites

**FIGURE 6:**
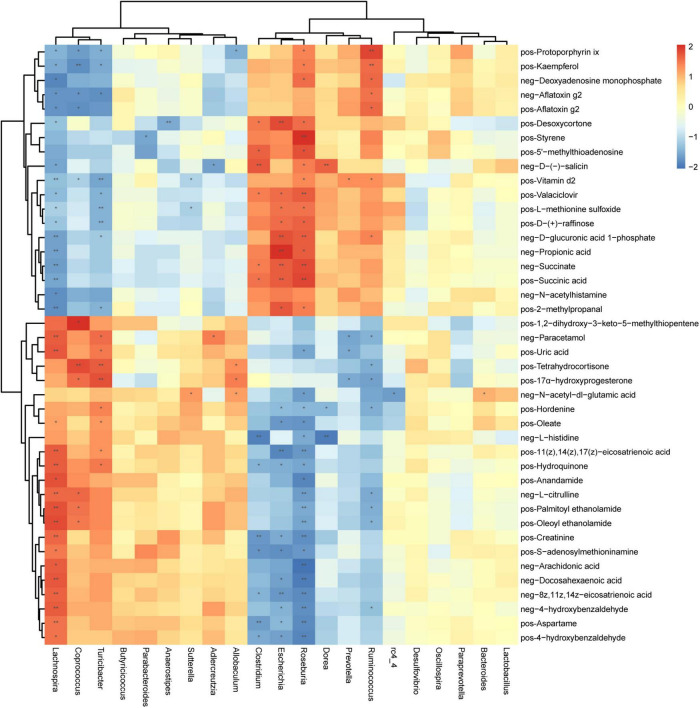
Correlation between microorganisms (genus level) and metabolites in feces samples of periodontitis. Each row represents a metabolite and each column represents a genus. Each grid represents the correlation coefficient between a metabolite and a genus. Red is positive correlation, and blue is negative correlation. *(*p* < 0.05), **(*p* < 0.01) Significant correlation between the genera and metabolites. pos, positive ions; neg, negative ions.

There is a strong correlation (*R* = 0.94) between microbial groups and the first component of differential metabolites (Supplementary Figure 9). To better understand the relationship between gut microbiota and fecal metabolites, we conducted a correlation analysis between genus-level microorganisms and key differential metabolites (Figure 6). *Roseburia*, *Lachnospira*, *Escherichia*, *Turicibacter*, *Ruminococcus*, *Clostridium*, and *Coprococcus* were the genera with the greatest correlations with differential fecal metabolites, and they have significantly correlated with 35, 34, 20, 17, 15, 13, and 11 metabolites, respectively (Supplementary Table 3). On the other hand, vitamin D2 and protoporphyrin IX had the most significant correlations with gut microbiota, which were significantly related to 7 and 6 different bacterial genera, respectively (Supplementary Table 4). Second, *N*-acetyl-DL-glutamic acid, D-glucuronic acid 1-phosphate, Paracetamol, D-(-)-salicin, L-methionine sulfoxide, Valaciclovir, Hordenine, Hydroquinone, Kaempferol, Desoxycortone, and 17α-hydroxyprogesterone were significantly correlated with 5 different genera (Supplementary Table 4).

## Discussion

Our multi-omics analysis results confirmed that periodontitis can change the composition of the gut microbiota and the fecal metabolism spectrum. Although species richness (i.e., the number of OTUs) was slightly higher in the EP group, there was no significant difference in α-diversity between the two groups, which is consistent with previous studies (Kirst et al., 2015; Rajan et al., 2020). However, the results of different researchers on periodontitis are not completely consistent (Wei et al., 2019). Our results are consistent with a previous study (Huang et al., 2020), there were significant differences in β-diversity between periodontitis and control based on the weighted and unweighted UniFrac distance.

This study found that periodontitis alters gut microbiota composition. Previous studies found that compared to healthy individuals, *Firmicutes* increased in EP groups (Lourenςo et al., 2018; Huang et al., 2020). Our results are contrary to this, which may partly be due to the high degree of variation between individuals in the microbiome (Costello et al., 2009). *Porphyromonas gingivalis* is a common pathogen of periodontitis. Nakajima et al. found that oral *P. gingivalis* significantly reduced *Firmicutes* in the intestinal flora (Arimatsu et al., 2014; Nakajima et al., 2015). The decrease in *Firmicutes* in this study may be due to *P. gingivalis* in the EP group. At the genus level, this study showed that *Ruminococcus*, *Roseburia*, and *Escherichia* were enriched in rats with periodontitis, while the proportion of *Coprococcus*, *Turicibacter*, and *Lachnospira* was significantly reduced. Our single microbiome analysis results are comparable to the results of previous studies (Lourenςo et al., 2018; Li et al., 2021; Watanabe et al., 2021) to a large extent.

Function analysis showed that there were more than 30 KEGG pathways significantly different in the gut microbiota of periodontitis. Including ABC transporters, valine, leucine, and isoleucine biosynthesis, fatty acid degradation, and cell cycle-Caulobacter. Other researchers have found similar functional pathways in the subgingival microbiota of periodontitis (LaMonte et al., 2018; Ebersole et al., 2020). Deng et al. (2017) have shown that the enriched functional pathways of the periodontal pocket in chronic periodontitis include bacterial chemotaxis, flagellar assembly, and two-component system. And these pathways are also detected in our study. The results of this study are comparable and unique to the results of previous studies. Through the correlation analysis, we found it interesting that the genera with significant differences in periodontitis are usually significantly correlated with more metabolites, such as *Roseburia*, *Lachnospira*, *Escherichia*, *Turicibacter*, and *Ruminococcus*. The genera with the same changing trend in EP tended to have a similar correlation with certain metabolites. *Clostridium*, which did not show any significant difference in the EP group, showed a significant correlation with fecal metabolites. It is suggested that there are complex connections between bacterial communities and metabolites.

More and more research evidence shows that the changes of gut microbiota affect the levels of metabolites, leading to the development of insulin resistance, atherosclerosis, and other diseases (Lin and Zhang, 2017; Canfora et al., 2019; Verhaar et al., 2020). Chen et al. (2021) showed that compared with healthy controls, periodontitis and high-fat diet had a significant impact on gingival metabolome, and experiments verified that high-fat diet had a superimposed effect on metabolome such as arginine metabolism in the presence of periodontitis. A recent animal study suggested that the periodontal pathogen *P. gingivalis* may increase insulin resistance and circulating branched-chain amino acids elevation through its metabolic activity (Tian et al., 2020). Komazaki et al. (2017) investigated the possible mechanism of periodontitis and non-alcoholic fatty liver by administering periodontal pathogens bacteria (*Aggregatibacteractino mycetemcomitans*) to rats, and found that it was mainly through fatty acid biosynthesis and fatty acid degradation pathways.

Growing research suggests that periodontitis may influence the progression of other diseases by altering gut microbiota and metabolism. It is reported that the gut microbiota affects the host’s metabolome (Visconti et al., 2019). We found that periodontitis increased gut bacteria *Ruminococcus.* Pernigoni et al. (2021) found that *Ruminococcus gnavus* converts the metabolites pregnenolone and hydroxypregnenolone to dehydroepiandrosterone and testosterone, regulates the steroid hormone biosynthesis pathway, thereby promoting the growth of prostate cancer. Studies have shown that benign prostatic hyperplasia has a significant impact on gut microbes and metabolism, and differential metabolites are mainly enriched in the steroid hormone biosynthesis pathways (Li et al., 2022). Periodontitis is a risk factor for prostate diseases, and animal experiments have found that periodontitis can promote the progression of benign prostatic hyperplasia (Fang et al., 2021a). However, the mechanism of interaction between periodontitis and prostate diseases has not been fully elucidated so far. We speculate that gut microbiota disturbances affecting metabolism may be one reason for periodontitis affecting prostate disease. The results of this study may provide some directions for future research, while the speculation still needed further study to confirm. If the hypothesis that periodontitis may influence other diseases by “oral-gut axis” be confirmed in the future, it can strengthen the maintenance of periodontal health to decrease the risk of systemic diseases.

In conclusion, this study not only provides the catalog of fecal metabolites that may reflect the metabolic changes in periodontitis but also illustrates the correlation analysis and related pathways between gut microbiota and fecal metabolites. The advantage of our study is that, compared to single-omics studies, the use of multi-omics methods provides a rich and complementary understanding of periodontitis and helps researchers better understand the disease. This study does have some limitations at present. First, the sample size of the study is small, and more samples need to be used for further verification research. Second, there are large differences in the gut microbiota between individuals. Overall, this multi-omics study systematically analyzes the effects of periodontitis on gut microbiota, fecal metabolites, and their enrichment pathways, which has great potential to help understand and provides a direction for further analysis of the potential mechanisms of periodontitis involved in other diseases.

## Data Availability Statement

The datasets presented in this study can be found in online repositories. The names of the repository/repositories and accession number(s) can be found in the article/Supplementary Material.

## Ethics Statement

The animal study was reviewed and approved by the Animal Ethics Committee of Wuhan University (IACUC 2018119).

## Author Contributions

LW contributed to design, data acquisition, and analysis and drafted and critically revised the manuscript. J-YN and JH contributed to design and data interpretation and critically revised the manuscript. TD and CL contributed to data acquisition and analysis and critically revised the manuscript. CF and W-ZX contributed to conception, design, and data interpretation, and critically revised the manuscript. S-YW contributed to data analysis and interpretation and drafted and critically revised the manuscript. X-TZ contributed to conception and design and critically revised the manuscript. All authors gave final approval and agreed to be accountable for all aspects of the work.

## Conflict of Interest

The authors declare that the research was conducted in the absence of any commercial or financial relationships that could be construed as a potential conflict of interest.

## Publisher’s Note

All claims expressed in this article are solely those of the authors and do not necessarily represent those of their affiliated organizations, or those of the publisher, the editors and the reviewers. Any product that may be evaluated in this article, or claim that may be made by its manufacturer, is not guaranteed or endorsed by the publisher.
