# Supplementary material for: Alterations and Correlations of Gut Microbiota and Fecal Metabolome Characteristics in Experimental Periodontitis Rats
Source: Front Microbiol. 2022 Apr 14;13:865191. doi: 10.3389/fmicb.2022.865191 (PMC9048259; doi:10.3389/fmicb.2022.865191)
Supplement: Supplementary file 1 [file Data_Sheet_1.docx]

**Alterations and Correlations of Gut Microbiota and Fecal Metabolome Characteristics in Experimental Periodontitis Rats**

Lan Wu^1,2^, Jie Han^3^, Jia-Yan Nie^3^, Tong Deng^1^, Cheng Li^2^, Cheng Fang^1^, Wen-Zhong Xie^4^, Shuang-Ying Wang^1^* and Xian-Tao Zeng^1^*

1 Center for Evidence-Based and Translational Medicine, Zhongnan Hospital of Wuhan University, Wuhan, China,

2 Department of Stomatology, Zhongnan Hospital of Wuhan University, Wuhan, China,

3 Department of Gastroenterology, Zhongnan Hospital of Wuhan University, Wuhan, China,

4 Department of Stomatology, Kaifeng University Health Science Center, Kaifeng, China

**Supplementary**

**1. Materials and methods**

**2. Supplementary Figure 1.** Diversity analysis of EP and Control group.

**3. Supplementary Figure 2**. Significantly different gut microbiota at phylum level in EP compared with Control.

**4. Supplementary Figure 3.** Linear discriminant analysis coupled with effect size (LEfSe) analysis showing bacteria that were altered between the EP and Control.

**5. Supplementary Figure 4.** Differentially enriched KEGG functions pathways (level 2) between EP and Control by PICRUST2 analysis.

**6.** **Supplementary Figure 5.** The base peak chromatogram of LC-MS experiments in positive ion modes.

**7. Supplementary Figure 6.** The base peak chromatogram of LC-MS experiments in negative ion modes.

**8. Supplementary Figure 7.** Periodontitis leads to changes in metabolic pathways which annotated by significant difference positive ion metabolite.

**9. Supplementary Figure 8.** Periodontitis leads to changes in metabolic pathways which annotated by significant difference negative ion metabolite.

**10. Supplementary Figure 9.** Scatter plot of correlation between differential metabolites and microbial groups.

**11. Supplementary Table 1.** The key fecal metabolites which significantly different in periodontitis compared with control and involved in the KEGG functional pathway.

**12.** **Supplementary Table 2.** Enrichment pathways of key fecal metabolite.

**13. Supplementary Table 3.** Metabolites that are significantly associated with microorganisms

**14. Supplementary Table 4.** Microorganisms that are significantly associated with metabolites

**15. Supplementary reference**

**Materials and methods**

**Periodontitis Rat Model and Sample Collection**

Rats (weighting 300-350 g) were randomized divided into 2 groups using a computer based random order generator. Rats were kept in specific pathogen-free (SPF) conditions, with constant humidity (55±10%) and temperature (22±2°C) for 12-hour light/dark cycle. During our experiment, we strictly controlled the diet and living environment of the rats. All rats live in the same environment and conditions, including the sterilized food and filler, and all breeding practices are treated equally and simultaneously. Therefore, confounding factors such as diet, obesity and stress are eliminated. The statisticians responsible for the randomization process and the animal experimenters are the only ones who know the distribution of the groups, while the sequencing experimenters and data analysis statisticians are unaware of the experimental grouping. No animals or data were excluded.

**Untargeted metabolomics**

The mobile phase of the positive ion mode is an aqueous solution containing 0.1% formic acid (liquid A) and 100% methanol containing 0.1% formic acid (liquid B). The mobile phase of the negative ion mode is an aqueous solution containing 10 mM ammonium formate (liquid A) and 95% methanol containing 10 mM ammonium formate (liquid B). Chromatographic separation of samples was realized by gradient elution.

**16S rDNA gene amplicon sequence analysis pipeline**

Then the paired-end reads were added to the tags through the Fast Length Adjustment of Short reads program (FLASH, v1.2.11) (Magoč and Salzberg, 2011). UPARSE software (v7.0.1090) (Edgar, 2013) was used to cluster the tags into Operational taxonomic unit (OTU) sequence at the cutoff of 97 %. Then UCHIME (V4.2.40) (Edgar et al., 2011) was used to compare the chimeras generated by PCR amplification with the Gold database (v20110519) and remove them from OTU representative sequences. A total of 735 OTUs were finally inferred. Ribosomal Database Project (RDP) Classifier (v.1.9.1) software with a minimum confidence threshold of 0.6 was used to compare OTU representative sequences with Greengene database (V201305) for species annotation. The USEARCH_global (Edgar, 2010) was used to compare all Tags back to OTU to get the OTU abundance statistics table of each sample.

OTU-based α-diversity was estimated by calculating Chao1, observed OTUs, Shannon and coverage using QIIME1 (v1.8.0) software (Caporaso et al., 2010) (OTU table random sub-sampling 5 times, each sample from 1 to 50,000 readings, with 500 readings as the step size). The Wilcox Test was used to analyze the differences in various indicators between the two groups. β-diversity is estimated by calculating Bray Curtis dissimilarity (vegan package in R 3.1.1) and weighted UniFrac distance (QIIME, v1.80). Permutational MANOVA (PERMANOVA) which performed by the adonis function in the vegan package, was used to compare the differences in community structure between groups. The effect size of linear discriminant analysis (LEfSe) with LDA value of 3.0 was used to determine the differential abundance of OTU between control and PD (Segata et al., 2011). KEGG functions were predicted using the PICRUSt2 software (v2.2.0-b) (Douglas et al., 2020). The Wilcoxon rank-sum test method, using the Wilcox-test package in R (v3.4.1), was used to test the differences in microbial genus, phylum level and function.


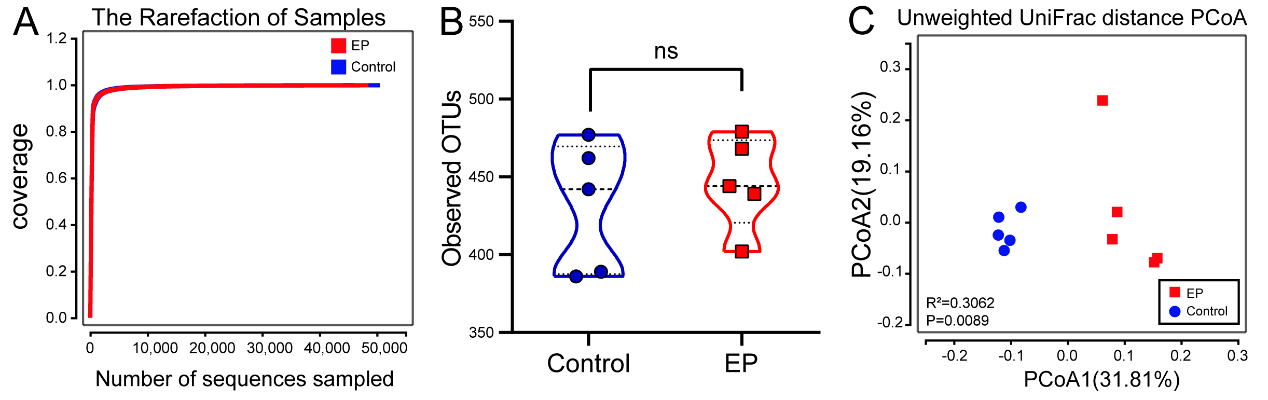


**Supplementary Figure 1.** Diversity analysis of EP and Control group. (A) Rarefaction curves of all samples. The coverage estimator of each group reached 99.8%, indicating that the sequencing depth is sufficient. (B) Observed OTUs is the index of the number of OTUs detected in all samples. (C) Principal Coordinate Analysis (PCoA) based on Unweighted Unifrac distance matrix. ns: no significance; EP: experimental periodontitis group; Control: healthy control group.


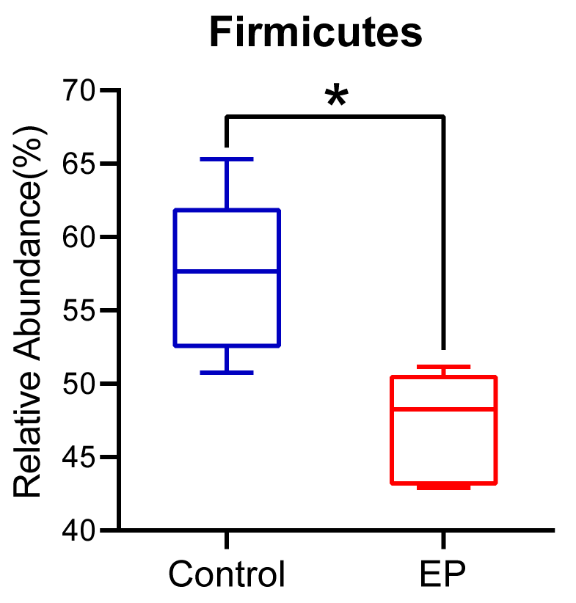


**Supplementary Figure 2.** Significantly different gut microbiota at phylum level in EP compared with Control. *p < 0.05. EP: experimental periodontitis group; Control: healthy control group.


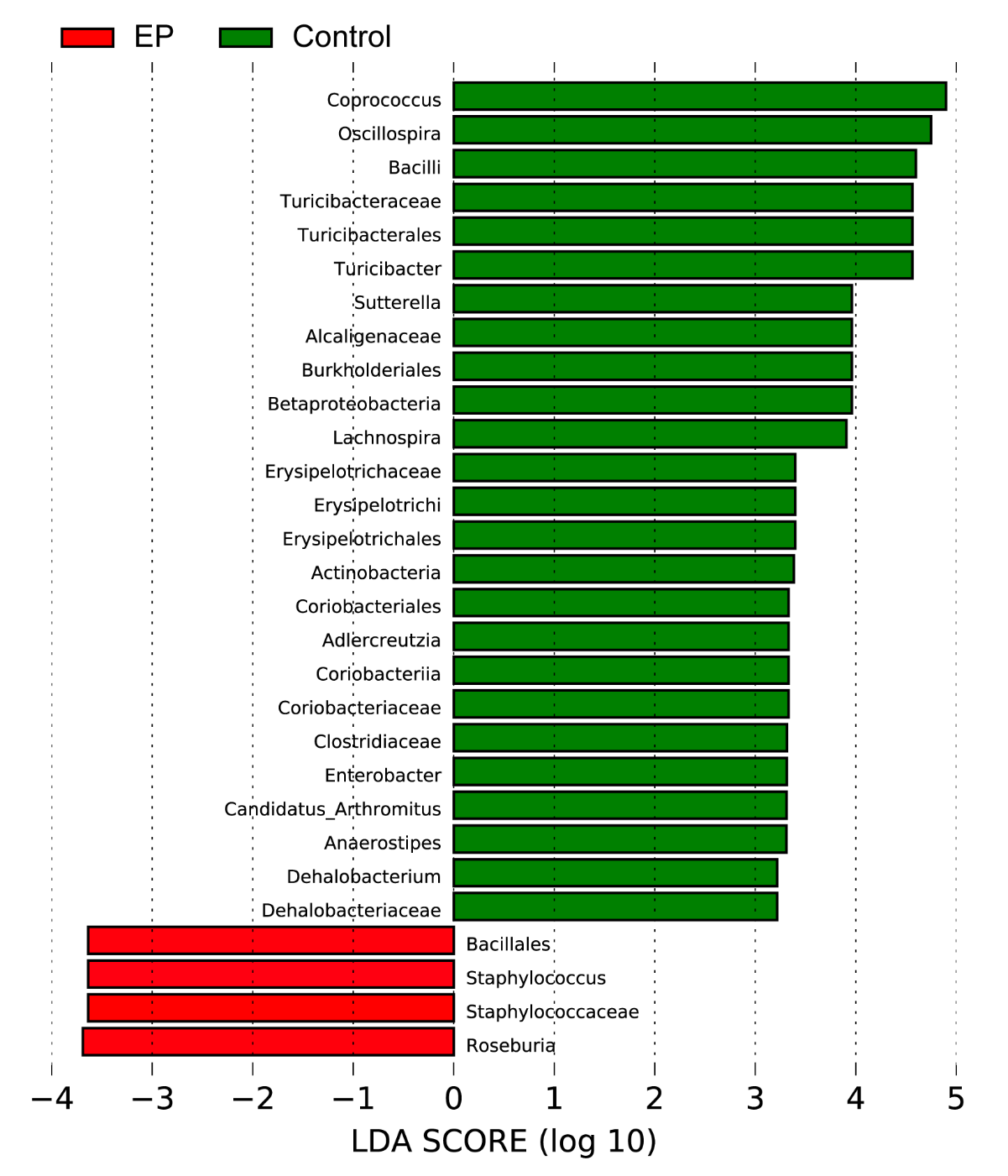


**Supplementary Figure 3.** Linear discriminant analysis coupled with effect size (LEfSe) analysis showing bacteria that were altered between the EP and Control. The number of bacterial branches in the EP group (red) and the Control group (green) was significantly different (P < 0.05) and had an LDA score > ±3. EP: experimental periodontitis group; Control: healthy control group.


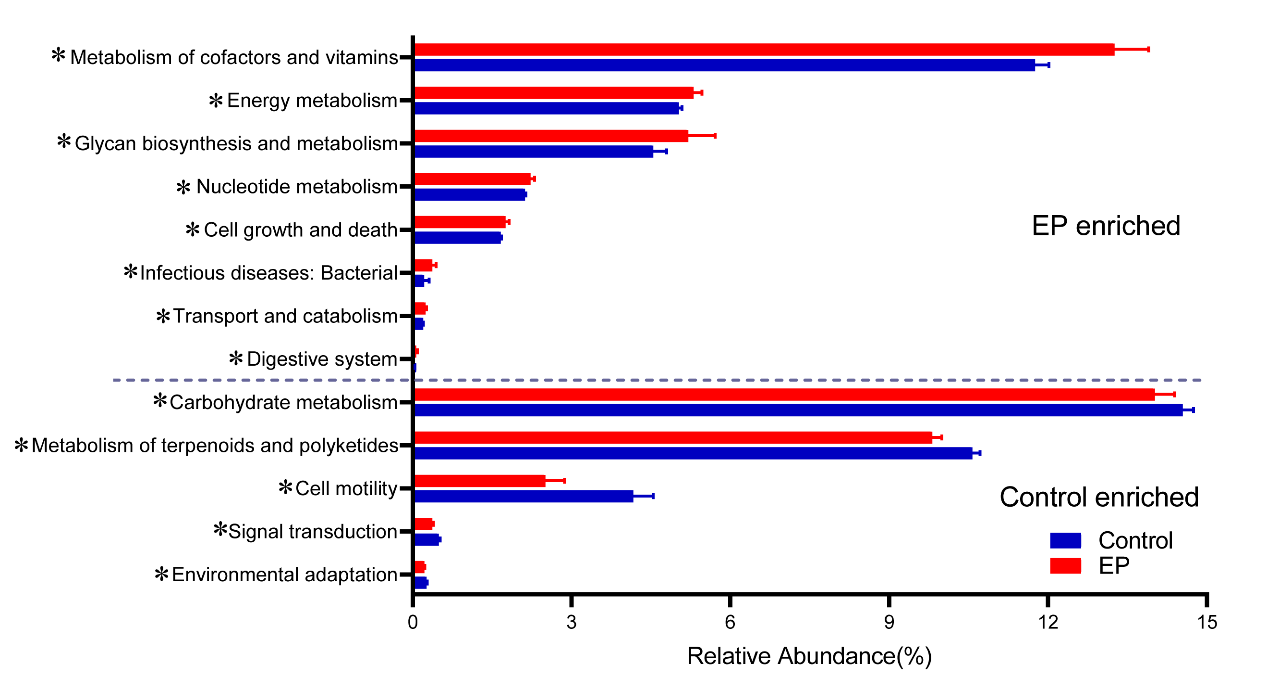


**Supplementary Figure 4.** Differentially enriched KEGG functions pathways (level 2) between EP and Control by PICRUST2 analysis. *p < 0.05. EP: experimental periodontitis group; Control: healthy control group.


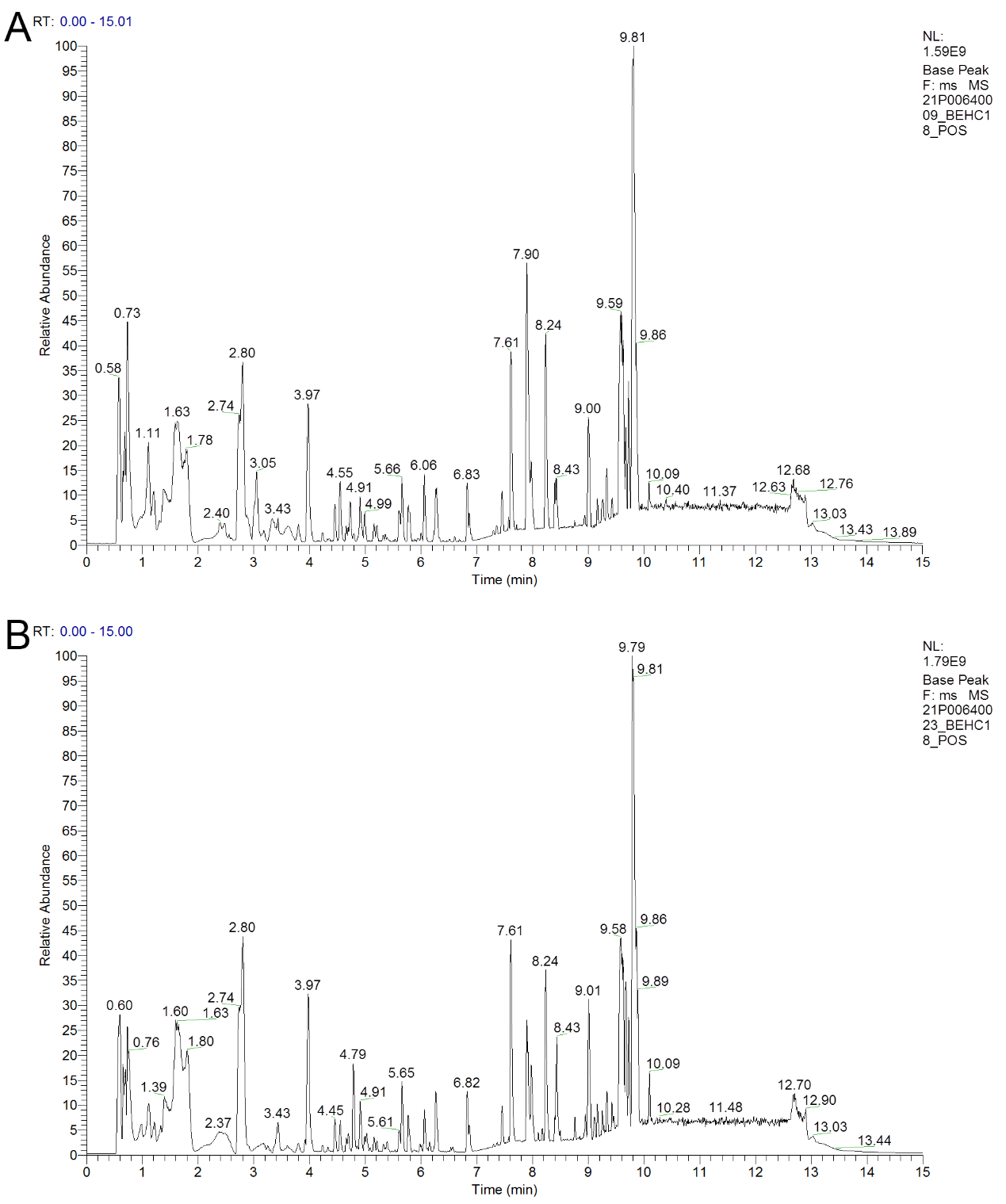


**Supplementary Figure 5.** The base peak chromatogram of LC-MS experiments in positive ion modes. A curve with time as abscissa and total ion intensity at each time point as ordinate. (A) the spectra of the experimental periodontitis group. (B) the spectra of the control group.


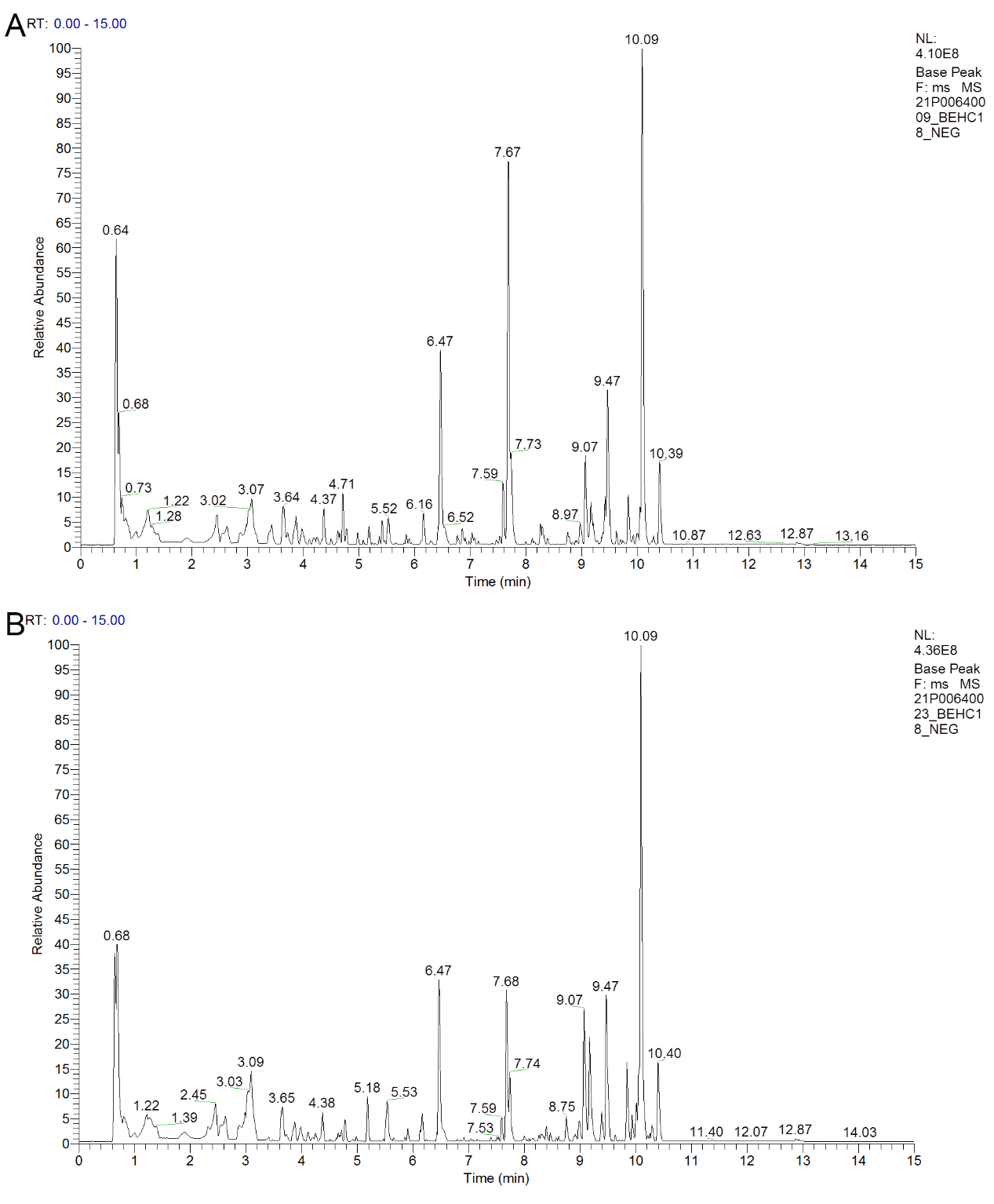


**Supplementary Figure 6.** The base peak chromatogram of LC-MS experiments in negative ion modes. A curve with time as abscissa and total ion intensity at each time point as ordinate. (A) the spectra of the experimental periodontitis group. (B) the spectra of the control group.


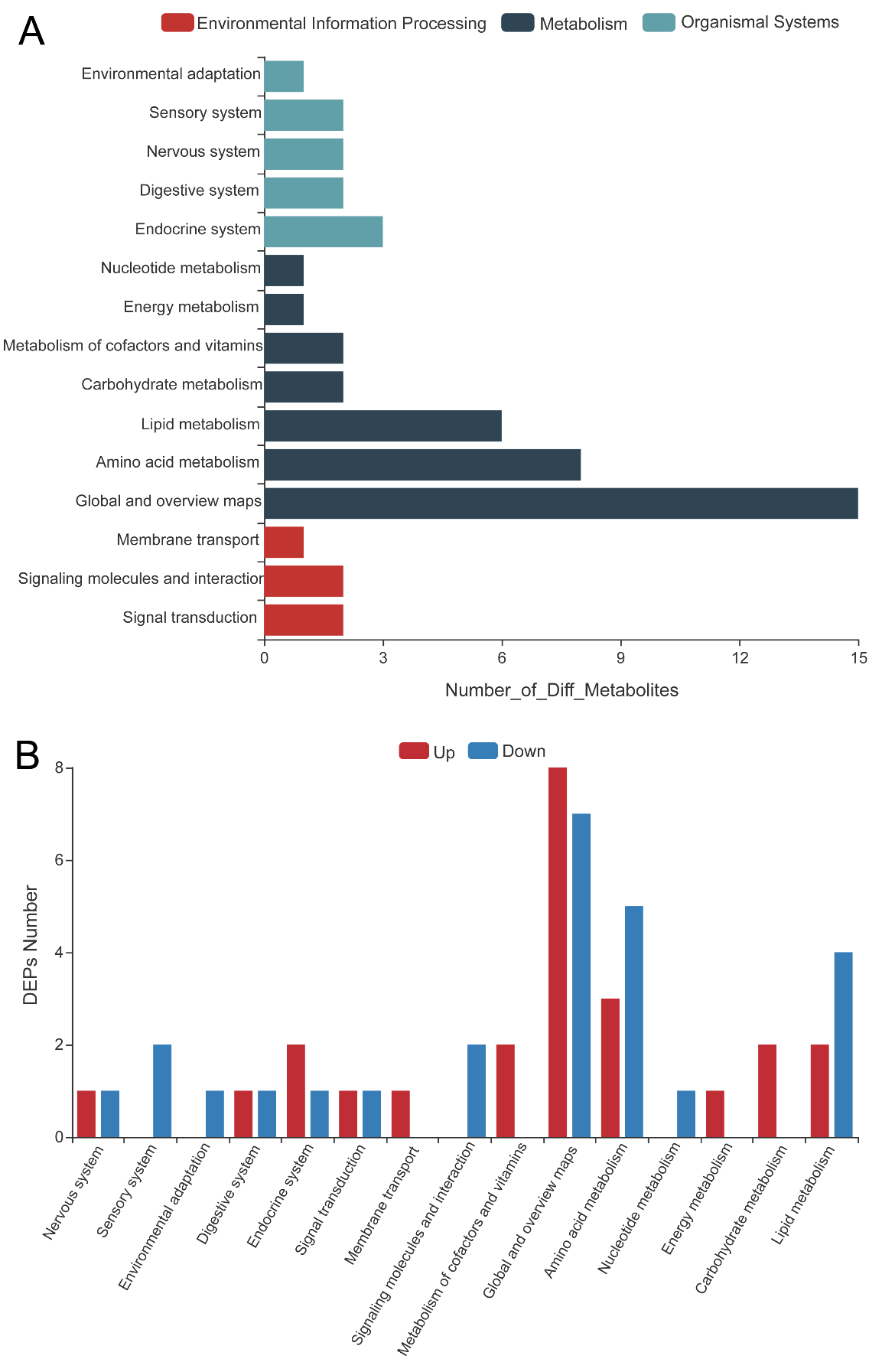


**Supplementary Figure 7.** Periodontitis leads to changes in metabolic pathways which annotated by significant difference positive ion metabolite. (A) KEGG pathway function annotation bar graph of positive ion: The X-axis represents the number of significant difference metabolite annotations, and the Y-axis represents the annotated KEGG Pathway. (B) Statistical up-regulation and down-regulation of pathway classification of differential metabolites.


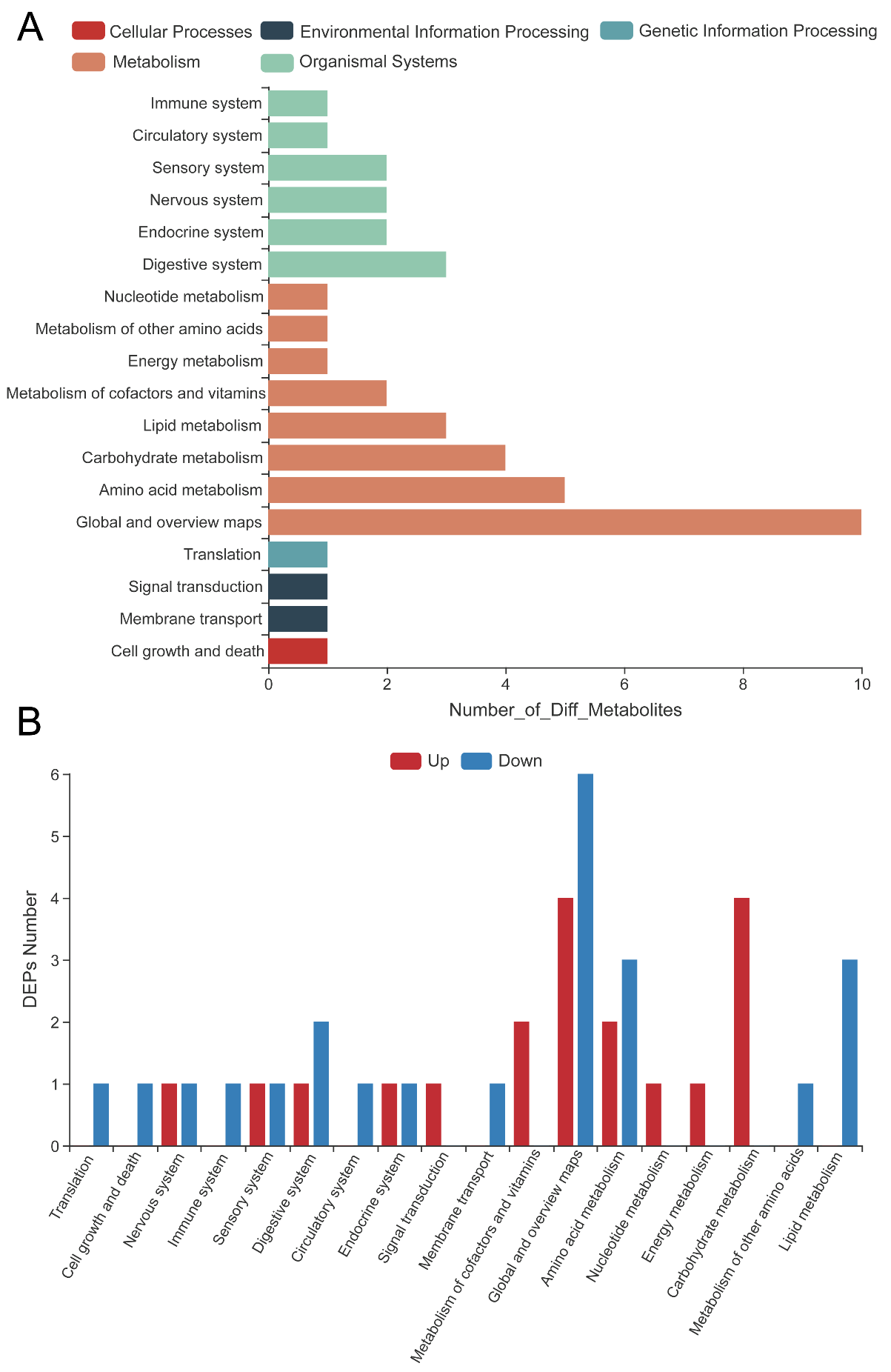


**Supplementary Figure 8.** Periodontitis leads to changes in metabolic pathways which annotated by significant difference negative ion metabolite. (A) KEGG pathway function annotation bar graph of negative ion: The X-axis represents the number of significant difference metabolite annotations, and the Y-axis represents the annotated KEGG Pathway. (B) Statistical up-regulation and down-regulation of pathway classification of differential metabolites.


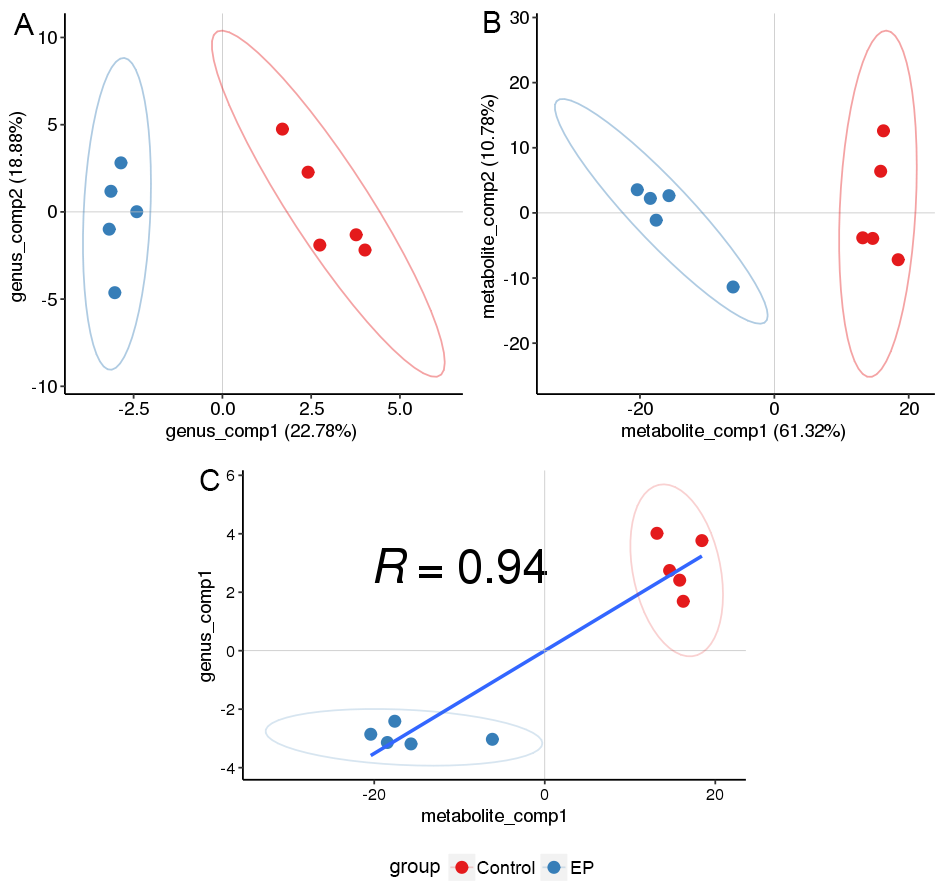


**Supplementary Figure 9.** Scatter plot of correlation between differential metabolites and microbial groups. (A) The component scatter plot of microbial genus group; (B) The component scatter diagram of differential metabolite. (C) Pearson correlation scatter diagram of the differential metabolite and the first component of microorganism group.

**Supplementary** **Table 1.** The key fecal metabolites which significantly different in periodontitis compared with control and involved in the KEGG functional pathway.

FC: means fold change, which was calculated as the average of PD relative to that of the controls.

Pvalue: means p-value obtained from Student’s t-test.

VIP: variable importance in projection.

| Name | FC | Pvalue | VIP | KEGG.ID | Pathway |
| --- | --- | --- | --- | --- | --- |
| Type: Positive-ion | | | | | |
| L-methionine sulfoxide | 42.228 | 0.0265 | 2.649 | C02989 | map00270 Cysteine and methionine metabolism; |
| Valaciclovir | 11.286 | 0.0493 | 2.018 | C07184 | map04976 Bile secretion; |
| Styrene | 8.5384 | 0.0315 | 1.739 | C07083 | map01100 Metabolic pathways; |
| D-(+)-raffinose | 3.4457 | 0.0268 | 1.702 | C00492 | map00052 Galactose metabolism; map01100 Metabolic pathways; map02010 ABC transporters; |
| Succinic acid | 3.1174 | 0.0102 | 1.298 | C00042 | map00020 Citrate cycle (TCA cycle); map00190 Oxidative phosphorylation; map00250 Alanine, aspartate and glutamate metabolism; map00310 Lysine degradation; map00350 Tyrosine metabolism; map00360 Phenylalanine metabolism; map00620 Pyruvate metabolism; map00630 Glyoxylate and dicarboxylate metabolism; map00640 Propanoate metabolism; map00650 Butanoate metabolism; map00760 Nicotinate and nicotinamide metabolism; map00920 Sulfur metabolism; map01100 Metabolic pathways; map01200 Carbon metabolism; map04024 cAMP signaling pathway; map04727 GABAergic synapse; map04922 Glucagon signaling pathway; map05230 Central carbon metabolism in cancer; |
| Desoxycortone | 2.9406 | 0.0366 | 1.509 | C03205 | map00140 Steroid hormone biosynthesis; map01100 Metabolic pathways; map04925 Aldosterone synthesis and secretion; |
| Protoporphyrin ix | 2.9178 | 0.0324 | 1.449 | C02191 | map00860 Porphyrin and chlorophyll metabolism; map01100 Metabolic pathways; |
| Kaempferol | 2.5973 | 0.0051 | 1.422 | C05903 | map01100 Metabolic pathways; |
| 5'-methylthioadenosine | 2.5854 | 0.0389 | 1.434 | C00170 | map00270 Cysteine and methionine metabolism; map01100 Metabolic pathways; |
| Aflatoxin g2 | 2.4062 | 0.0096 | 1.43 | C16754 | map01100 Metabolic pathways; |
| 2-methylpropanal | 2.1305 | 0.0193 | 1.253 | C03219 | map01100 Metabolic pathways; map01210 2-Oxocarboxylic acid metabolism; |
| Vitamin d2 | 2.0868 | 0.0279 | 1.034 | C05441 | map00100 Steroid biosynthesis; map01100 Metabolic pathways; |
| Uric acid | 0.5248 | 0.0134 | 1.059 | C00366 | map00230 Purine metabolism; map01100 Metabolic pathways; map04976 Bile secretion; |
| Tetrahydrocortisone | 0.4866 | 0.0251 | 1.286 | C05470 | map00140 Steroid hormone biosynthesis; |
| 11(z),14(z),17(z)-eicosatrienoic acid | 0.4856 | 0.0125 | 1.291 | C16522 | map01040 Biosynthesis of unsaturated fatty acids; |
| Oleate | 0.4773 | 0.0063 | 1.187 | C00712 | map00061 Fatty acid biosynthesis; map01040 Biosynthesis of unsaturated fatty acids; |
| 1,2-dihydroxy-3-keto-5-methylthiopentene | 0.4726 | 0.0389 | 1.331 | C15606 | map00270 Cysteine and methionine metabolism; map01100 Metabolic pathways; |
| S-adenosylmethioninamine | 0.4438 | 0.0167 | 1.456 | C01137 | map00270 Cysteine and methionine metabolism; map00330 Arginine and proline metabolism; map01100 Metabolic pathways; |
| Palmitoyl ethanolamide | 0.4408 | 0.0011 | 1.524 | C16512 | map04080 Neuroactive ligand-receptor interaction; |
| Anandamide | 0.4406 | 0.0336 | 1.203 | C11695 | map04080 Neuroactive ligand-receptor interaction; map04714 Thermogenesis; map04723 Retrograde endocannabinoid signaling; map04750 Inflammatory mediator regulation of TRP channels; |
| Hydroquinone | 0.3983 | 0.0128 | 1.083 | C00530 | map00350 Tyrosine metabolism; map01100 Metabolic pathways; |
| 17α-hydroxyprogesterone | 0.3887 | 0.0286 | 1.564 | C01176 | map00140 Steroid hormone biosynthesis; map01100 Metabolic pathways; map04913 Ovarian steroidogenesis; map04927 Cortisol synthesis and secretion; map04934 Cushing syndrome; |
| Oleoyl ethanolamide | 0.3548 | 0.0032 | 1.872 | C20792 | map04024 cAMP signaling pathway; |
| Aspartame | 0.3427 | 0.0147 | 1.409 | C11045 | map04742 Taste transduction; |
| Hordenine | 0.2918 | 0.0068 | 1.646 | C06199 | map00350 Tyrosine metabolism; map01100 Metabolic pathways; |
| 4-hydroxybenzaldehyde | 0.2697 | 0.0335 | 1.533 | C00633 | map01100 Metabolic pathways; |
| Creatinine | 0.1322 | 0.0144 | 4.134 | C00791 | map00330 Arginine and proline metabolism; map01100 Metabolic pathways; |
| Type: Negative-ion | | | | | |
| N-acetylhistamine | 5.6189 | 0.0314 | 1.59 | C05135 | map00340 Histidine metabolism; |
| D-glucuronic acid 1-phosphate | 3.3359 | 0.0013 | 1.692 | C05385 | map00040 Pentose and glucuronate interconversions; map00053 Ascorbate and aldarate metabolism; map00520 Amino sugar and nucleotide sugar metabolism; map01100 Metabolic pathways; |
| Propionic acid | 2.7145 | 0.0034 | 1.276 | C00163 | map00640 Propanoate metabolism; map00760 Nicotinate and nicotinamide metabolism; map01100 Metabolic pathways; map04973 Carbohydrate digestion and absorption; map04974 Protein digestion and absorption; |
| Succinate | 2.5717 | 0.0107 | 1.252 | C00042 | map00020 Citrate cycle (TCA cycle); map00190 Oxidative phosphorylation; map00250 Alanine, aspartate and glutamate metabolism; map00310 Lysine degradation; map00350 Tyrosine metabolism; map00360 Phenylalanine metabolism; map00620 Pyruvate metabolism; map00630 Glyoxylate and dicarboxylate metabolism; map00640 Propanoate metabolism; map00650 Butanoate metabolism; map00760 Nicotinate and nicotinamide metabolism; map00920 Sulfur metabolism; map01100 Metabolic pathways; map01200 Carbon metabolism; map04024 cAMP signaling pathway; map04727 GABAergic synapse; map04922 Glucagon signaling pathway; map05230 Central carbon metabolism in cancer; |
| D-(-)-salicin | 2.2776 | 0.0202 | 1.169 | C01451 | map00010 Glycolysis / Gluconeogenesis; map04742 Taste transduction; |
| Aflatoxin g2 | 2.2383 | 0.0225 | 1.357 | C16754 | map01100 Metabolic pathways; |
| Deoxyadenosine monophosphate | 2.0703 | 0.0236 | 1.351 | C00360 | map00230 Purine metabolism; map01100 Metabolic pathways; |
| N-acetyl-dl-glutamic acid | 0.606 | 0.0435 | 1.139 | C00624 | map00220 Arginine biosynthesis; map01100 Metabolic pathways; map01210 2-Oxocarboxylic acid metabolism; map01230 Biosynthesis of amino acids; |
| L-histidine | 0.5714 | 0.0467 | 1.055 | C00135 | map00340 Histidine metabolism; map00410 beta-Alanine metabolism; map00970 Aminoacyl-tRNA biosynthesis; map01100 Metabolic pathways; map01230 Biosynthesis of amino acids; map02010 ABC transporters; map04974 Protein digestion and absorption; map05230 Central carbon metabolism in cancer; |
| Arachidonic acid | 0.4981 | 0.0364 | 1.22 | C00219 | map00590 Arachidonic acid metabolism; map00591 Linoleic acid metabolism; map01040 Biosynthesis of unsaturated fatty acids; map01100 Metabolic pathways; map04216 Ferroptosis; map04217 Necroptosis; map04270 Vascular smooth muscle contraction; map04611 Platelet activation; map04664 Fc epsilon RI signaling pathway; map04666 Fc gamma R-mediated phagocytosis; map04723 Retrograde endocannabinoid signaling; map04726 Serotonergic synapse; map04730 Long-term depression; map04750 Inflammatory mediator regulation of TRP channels; map04912 GnRH signaling pathway; map04913 Ovarian steroidogenesis; map04921 Oxytocin signaling pathway; map04923 Regulation of lipolysis in adipocytes; map04925 Aldosterone synthesis and secretion; map05140 Leishmaniasis; map05146 Amoebiasis; |
| Docosahexaenoic acid | 0.4616 | 0.0101 | 1.471 | C06429 | map01040 Biosynthesis of unsaturated fatty acids; |
| Paracetamol | 0.4444 | 0.0478 | 1.296 | C06804 | map04976 Bile secretion; |
| L-citrulline | 0.4077 | 0.0187 | 1.464 | C00327 | map00220 Arginine biosynthesis; map01100 Metabolic pathways; map01230 Biosynthesis of amino acids; |
| 4-hydroxybenzaldehyde | 0.3093 | 0.0407 | 1.539 | C00633 | map01100 Metabolic pathways; |
| 8z,11z,14z-eicosatrienoic acid | 0.3092 | 0.0112 | 1.874 | C03242 | map00591 Linoleic acid metabolism; map01040 Biosynthesis of unsaturated fatty acids; map01100 Metabolic pathways; |

**Supplementary Table 2.** Enrichment pathways of key fecal metabolite.

| Pathway | Pathway.ID | Count | Count.All | RichFactor | Pvalue | Metabolites |
| --- | --- | --- | --- | --- | --- | --- |
| Type: Positive-ion | | | | | | |
| Cysteine and methionine metabolism | map00270 | 4 | 61 | 0.0655738 | 6.64E-05 | L-methionine sulfoxide;S-adenosylmethioninamine;5'-methylthioadenosine;1,2-dihydroxy-3-keto-5-methylthiopentene |
| Metabolic pathways | map01100 | 14 | 1706 | 0.0082063 | 0.002431 | Creatinine;S-adenosylmethioninamine;Uric acid;Succinic acid;Protoporphyrin ix;Vitamin d2;Styrene;Hydroquinone;5'-methylthioadenosine;1,2-dihydroxy-3-keto-5-methylthiopentene;4-hydroxybenzaldehyde;Kaempferol;17α-hydroxyprogesterone;Desoxycortone |
| Tyrosine metabolism | map00350 | 3 | 78 | 0.0384615 | 0.002732 | Hordenine;Succinic acid;Hydroquinone |
| cAMP signaling pathway | map04024 | 2 | 25 | 0.08 | 0.003572 | Succinic acid;Oleoyl ethanolamide |
| Steroid hormone biosynthesis | map00140 | 3 | 99 | 0.030303 | 0.005341 | Tetrahydrocortisone;17α-hydroxyprogesterone;Desoxycortone |
| Neuroactive ligand-receptor interaction | map04080 | 2 | 52 | 0.0384615 | 0.014841 | Palmitoyl ethanolamide;Anandamide |
| Biosynthesis of unsaturated fatty acids | map01040 | 2 | 74 | 0.027027 | 0.028755 | 11(z),14(z),17(z)-eicosatrienoic acid;Oleate |
| Arginine and proline metabolism | map00330 | 2 | 78 | 0.025641 | 0.031681 | Creatinine;S-adenosylmethioninamine |
| GABAergic synapse | map04727 | 1 | 9 | 0.1111111 | 0.031684 | Succinic acid |
| Cortisol synthesis and secretion | map04927 | 1 | 12 | 0.0833333 | 0.042024 | 17α-hydroxyprogesterone |
| Bile secretion | map04976 | 2 | 97 | 0.0206186 | 0.047055 | Valaciclovir;Uric acid |
| Type: Negative-ion | | | | | | |
| Metabolic pathways | map01100 | 10 | 1706 | 0.0058617 | 0.000121 | Propionic acid;Succinate;N-acetyl-dl-glutamic acid;D-glucuronic acid 1-phosphate;L-citrulline;L-histidine;Arachidonic acid;8z,11z,14z-eicosatrienoic acid;4-hydroxybenzaldehyde;Deoxyadenosine monophosphate |
| Biosynthesis of unsaturated fatty acids | map01040 | 3 | 74 | 0.0405405 | 0.000188 | Docosahexaenoic acid;Arachidonic acid;8z,11z,14z-eicosatrienoic acid |
| Arginine biosynthesis | map00220 | 2 | 23 | 0.0869565 | 0.000549 | N-acetyl-dl-glutamic acid;L-citrulline |
| Linoleic acid metabolism | map00591 | 2 | 28 | 0.0714286 | 0.000816 | Arachidonic acid;8z,11z,14z-eicosatrienoic acid |
| Biosynthesis of amino acids | map01230 | 3 | 128 | 0.0234375 | 0.000936 | N-acetyl-dl-glutamic acid;L-citrulline;L-histidine |
| Protein digestion and absorption | map04974 | 2 | 47 | 0.0425532 | 0.002293 | Propionic acid;L-histidine |
| Histidine metabolism | map00340 | 2 | 47 | 0.0425532 | 0.002293 | L-histidine;N-acetylhistamine |
| Propanoate metabolism | map00640 | 2 | 48 | 0.0416667 | 0.00239 | Propionic acid;Succinate |
| Nicotinate and nicotinamide metabolism | map00760 | 2 | 55 | 0.0363636 | 0.003126 | Propionic acid;Succinate |
| GnRH signaling pathway | map04912 | 1 | 6 | 0.1666667 | 0.009055 | Arachidonic acid |
| Fc gamma R-mediated phagocytosis | map04666 | 1 | 8 | 0.125 | 0.012056 | Arachidonic acid |
| Long-term depression | map04730 | 1 | 9 | 0.1111111 | 0.013553 | Arachidonic acid |
| GABAergic synapse | map04727 | 1 | 9 | 0.1111111 | 0.013553 | Succinate |
| Necroptosis | map04217 | 1 | 10 | 0.1 | 0.015047 | Arachidonic acid |
| Fc epsilon RI signaling pathway | map04664 | 1 | 11 | 0.0909091 | 0.01654 | Arachidonic acid |
| Oxytocin signaling pathway | map04921 | 1 | 12 | 0.0833333 | 0.018031 | Arachidonic acid |
| Platelet activation | map04611 | 1 | 14 | 0.0714286 | 0.021005 | Arachidonic acid |
| Regulation of lipolysis in adipocytes | map04923 | 1 | 14 | 0.0714286 | 0.021005 | Arachidonic acid |
| Oxidative phosphorylation | map00190 | 1 | 16 | 0.0625 | 0.023971 | Succinate |
| Vascular smooth muscle contraction | map04270 | 1 | 16 | 0.0625 | 0.023971 | Arachidonic acid |
| Retrograde endocannabinoid signaling | map04723 | 1 | 19 | 0.0526316 | 0.028403 | Arachidonic acid |
| Citrate cycle (TCA cycle) | map00020 | 1 | 20 | 0.05 | 0.029876 | Succinate |
| Aldosterone synthesis and secretion | map04925 | 1 | 22 | 0.0454545 | 0.032816 | Arachidonic acid |
| Ovarian steroidogenesis | map04913 | 1 | 24 | 0.0416667 | 0.035747 | Arachidonic acid |
| cAMP signaling pathway | map04024 | 1 | 25 | 0.04 | 0.03721 | Succinate |
| Glucagon signaling pathway | map04922 | 1 | 26 | 0.0384615 | 0.03867 | Succinate |
| Carbohydrate digestion and absorption | map04973 | 1 | 27 | 0.037037 | 0.040128 | Propionic acid |
| Alanine, aspartate and glutamate metabolism | map00250 | 1 | 28 | 0.0357143 | 0.041584 | Succinate |
| Ferroptosis | map04216 | 1 | 29 | 0.0344828 | 0.043038 | Arachidonic acid |
| Glycolysis / Gluconeogenesis | map00010 | 1 | 31 | 0.0322581 | 0.04594 | D-(-)-salicin |
| Pyruvate metabolism | map00620 | 1 | 31 | 0.0322581 | 0.04594 | Succinate |
| Taste transduction | map04742 | 1 | 32 | 0.03125 | 0.047387 | D-(-)-salicin |
| beta-Alanine metabolism | map00410 | 1 | 32 | 0.03125 | 0.047387 | L-histidine |
| Sulfur metabolism | map00920 | 1 | 33 | 0.030303 | 0.048832 | Succinate |

**Supplementary Table 3.** Metabolites that are significantly associated with microorganisms

| Microbiota (genus) | Number | Significantly related metabolites |
| --- | --- | --- |
| *Roseburia* | 35 | Propionic acid; Succinate; N-acetyl-dl-glutamic acid; D-glucuronic acid 1-phosphate; L-citrulline; L-histidine; Docosahexaenoic acid; Arachidonic acid; 8z,11z,14z-eicosatrienoic acid; neg-4-hydroxybenzaldehyde; D-(-)-salicin; Deoxyadenosine monophosphate; L-methionine sulfoxide; D-(+)-raffinose; Valaciclovir; Hordenine; Creatinine; S-adenosylmethioninamine; Uric acid; Succinic acid; 11(z),14(z),17(z)-eicosatrienoic acid; Protoporphyrin IX; Vitamin D2; Styrene; Hydroquinone; 5'-methylthioadenosine; Aspartame; 4-hydroxybenzaldehyde; Kaempferol; 2-methylpropanal; Desoxycortone; Palmitoyl ethanolamide; Oleoyl ethanolamide; Oleate; Anandamide |
| *Lachnospira* | 34 | Propionic acid; Succinate; D-glucuronic acid 1-phosphate; L-citrulline; N-acetylhistamine; Docosahexaenoic acid; Arachidonic acid; 8z,11z,14z-eicosatrienoic acid; Paracetamol; neg-4-hydroxybenzaldehyde; D-(-)-salicin; Deoxyadenosine monophosphate; neg-Aflatoxin g2; L-methionine sulfoxide; D-(+)-raffinose; Valaciclovir; Creatinine; S-adenosylmethioninamine; Uric acid; Succinic acid; 11(z),14(z),17(z)-eicosatrienoic acid; Protoporphyrin IX; Vitamin D2; Hydroquinone; Aspartame; 4-hydroxybenzaldehyde; Kaempferol; 2-methylpropanal; Aflatoxin g2; Desoxycortone; Palmitoyl ethanolamide; Oleoyl ethanolamide; Oleate; Anandamide |
| *Escherichia* | 20 | Propionic acid; Succinate; D-glucuronic acid 1-phosphate; Docosahexaenoic acid; 8z,11z,14z-eicosatrienoic acid; neg-4-hydroxybenzaldehyde; L-methionine sulfoxide; D-(+)-raffinose; Valaciclovir; Hordenine; Creatinine; S-adenosylmethioninamine; Succinic acid; 11(z),14(z),17(z)-eicosatrienoic acid; Hydroquinone; Aspartame; 4-hydroxybenzaldehyde; 2-methylpropanal; Desoxycortone; Oleate |
| *Turicibacter* | 17 | D-glucuronic acid 1-phosphate; Paracetamol; neg-Aflatoxin g2; L-methionine sulfoxide; D-(+)-raffinose; Valaciclovir; Hordenine; Uric acid; 11(z),14(z),17(z)-eicosatrienoic acid; Protoporphyrin IX; Vitamin D2; Hydroquinone; Kaempferol; Tetrahydrocortisone; 2-methylpropanal; 17-hydroxyprogesterone; Oleate |
| *Ruminococcus* | 15 | D-glucuronic acid 1-phosphate; L-citrulline; Paracetamol; neg-4-hydroxybenzaldehyde; Deoxyadenosine monophosphate; neg-Aflatoxin g2; Hordenine; Protoporphyrin IX; Vitamin D2; Kaempferol; Tetrahydrocortisone; Aflatoxin g2; 17-hydroxyprogesterone; Palmitoyl ethanolamide; Oleoyl ethanolamide |
| *Clostridium* | 13 | Succinate; L-histidine; 8z,11z,14z-eicosatrienoic acid; D-(-)-salicin; Valaciclovir; Creatinine; S-adenosylmethioninamine; Succinic acid; Hydroquinone; 5'-methylthioadenosine; Aspartame; 4-hydroxybenzaldehyde; Desoxycortone |
| *Coprococcus* | 11 | L-citrulline; neg-Aflatoxin g2; Protoporphyrin IX; Vitamin D2; 1,2-dihydroxy-3-keto-5-methylthiopentene; Kaempferol; Tetrahydrocortisone; Aflatoxin g2; 17-hydroxyprogesterone; Palmitoyl ethanolamide; Oleoyl ethanolamide |
| *Allobaculum* | 4 | N-acetyl-dl-glutamic acid; Protoporphyrin IX; Tetrahydrocortisone; 17-hydroxyprogesterone |
| *Prevotella* | 4 | Paracetamol; Uric acid; Vitamin D2; 17-hydroxyprogesterone |
| *Dorea* | 3 | L-histidine; D-(-)-salicin; Hordenine |
| *Sutterella* | 3 | N-acetyl-dl-glutamic acid; L-methionine sulfoxide; Vitamin D2 |
| *Adlercreutzia* | 2 | Paracetamol; D-(-)-salicin |
| *Anaerostipes* | 1 | Desoxycortone |
| *Bacteroides* | 1 | N-acetyl-dl-glutamic acid |
| *Parabacteroides* | 1 | Styrene |
| *rc4_4* | 1 | N-acetyl-dl-glutamic acid |
| *Butyricicoccus* | 0 | NA |
| *Desulfovibrio* | 0 | NA |
| *Lactobacillus* | 0 | NA |
| *Oscillospira* | 0 | NA |
| *Paraprevotella* | 0 | NA |

**Supplementary Table 4.** Microorganisms that are significantly associated with metabolites

| Metabolites | Number | Significantly related microbiota (genus) |
| --- | --- | --- |
| Vitamin D2 | 7 | *Coprococcus; Lachnospira; Prevotella; Roseburia; Ruminococcus; Sutterella; Turicibacter* |
| Protoporphyrin IX | 6 | *Allobaculum; Coprococcus; Lachnospira; Roseburia; Ruminococcus; Turicibacter* |
| N-acetyl-dl-glutamic acid | 5 | *Allobaculum; Bacteroides; Roseburia; Sutterella; rc4_4* |
| D-glucuronic acid 1-phosphate | 5 | *Escherichia; Lachnospira; Roseburia; Ruminococcus; Turicibacter* |
| Paracetamol | 5 | *Adlercreutzia; Lachnospira; Prevotella; Ruminococcus; Turicibacter* |
| D-(-)-salicin | 5 | *Adlercreutzia; Clostridium; Dorea; Lachnospira; Roseburia* |
| L-methionine sulfoxide | 5 | *Escherichia; Lachnospira; Roseburia; Sutterella; Turicibacter* |
| Valaciclovir | 5 | *Clostridium; Escherichia; Lachnospira; Roseburia; Turicibacter* |
| Hordenine | 5 | *Dorea; Escherichia; Roseburia; Ruminococcus; Turicibacter* |
| Hydroquinone | 5 | *Clostridium; Escherichia; Lachnospira; Roseburia; Turicibacter* |
| Kaempferol | 5 | *Coprococcus; Lachnospira; Roseburia; Ruminococcus; Turicibacter* |
| 17α-hydroxyprogesterone | 5 | *Allobaculum; Coprococcus; Prevotella; Ruminococcus; Turicibacter* |
| Desoxycortone | 5 | *Anaerostipes; Clostridium; Escherichia; Lachnospira; Roseburia* |
| Succinate | 4 | *Clostridium; Escherichia; Lachnospira; Roseburia* |
| L-citrulline | 4 | *Coprococcus; Lachnospira; Roseburia; Ruminococcus* |
| 8z,11z,14z-eicosatrienoic acid | 4 | *Clostridium; Escherichia; Lachnospira; Roseburia* |
| neg-4-hydroxybenzaldehyde | 4 | *Escherichia; Lachnospira; Roseburia; Ruminococcus* |
| neg-Aflatoxin g2 | 4 | *Coprococcus; Lachnospira; Ruminococcus; Turicibacter* |
| D-(+)-raffinose | 4 | *Escherichia; Lachnospira; Roseburia; Turicibacter* |
| Creatinine | 4 | *Clostridium; Escherichia; Lachnospira; Roseburia* |
| S-adenosylmethioninamine | 4 | *Clostridium; Escherichia; Lachnospira; Roseburia* |
| Uric acid | 4 | *Lachnospira; Prevotella; Roseburia; Turicibacter* |
| Succinic acid | 4 | *Clostridium; Escherichia; Lachnospira; Roseburia* |
| 11(z),14(z),17(z)-eicosatrienoic acid | 4 | *Escherichia; Lachnospira; Roseburia; Turicibacter* |
| Aspartame | 4 | *Clostridium; Escherichia; Lachnospira; Roseburia* |
| 4-hydroxybenzaldehyde | 4 | *Clostridium; Escherichia; Lachnospira; Roseburia* |
| Tetrahydrocortisone | 4 | *Allobaculum; Coprococcus; Ruminococcus; Turicibacter* |
| 2-methylpropanal | 4 | *Escherichia; Lachnospira; Roseburia; Turicibacter* |
| Palmitoyl ethanolamide | 4 | *Coprococcus; Lachnospira; Roseburia; Ruminococcus* |
| Oleoyl ethanolamide | 4 | *Coprococcus; Lachnospira; Roseburia; Ruminococcus* |
| Oleate | 4 | *Escherichia; Lachnospira; Roseburia; Turicibacter* |
| Propionic acid | 3 | *Escherichia; Lachnospira; Roseburia* |
| L-histidine | 3 | *Clostridium; Dorea; Roseburia* |
| Docosahexaenoic acid | 3 | *Escherichia; Lachnospira; Roseburia* |
| Deoxyadenosine monophosphate | 3 | *Lachnospira; Roseburia; Ruminococcus* |
| Aflatoxin g2 | 3 | *Coprococcus; Lachnospira; Ruminococcus* |
| Arachidonic acid | 2 | *Lachnospira; Roseburia* |
| Styrene | 2 | *Parabacteroides; Roseburia* |
| 5'-methylthioadenosine | 2 | *Clostridium; Roseburia* |
| Anandamide | 2 | *Lachnospira; Roseburia* |
| N-acetylhistamine | 1 | *Lachnospira* |
| 1,2-dihydroxy-3-keto-5-methylthiopentene | 1 | *Coprococcus* |

**Supplementary Reference**

Caporaso, J.G., Kuczynski, J., Stombaugh, J., Bittinger, K., Bushman, F.D., Costello, E.K., et al. (2010). QIIME allows analysis of high-throughput community sequencing data. *Nat Methods* 7(5)**,** 335-336. doi: 10.1038/nmeth.f.303.

Douglas, G.M., Maffei, V.J., Zaneveld, J.R., Yurgel, S.N., Brown, J.R., Taylor, C.M., et al. (2020). PICRUSt2 for prediction of metagenome functions. *Nat Biotechnol* 38(6)**,** 685-688. doi: 10.1038/s41587-020-0548-6.

Edgar, R.C. (2010). Search and clustering orders of magnitude faster than BLAST. *Bioinformatics* 26(19)**,** 2460-2461. doi: 10.1093/bioinformatics/btq461.

Edgar, R.C. (2013). UPARSE: highly accurate OTU sequences from microbial amplicon reads. *Nat Methods* 10(10)**,** 996-998. doi: 10.1038/nmeth.2604.

Edgar, R.C., Haas, B.J., Clemente, J.C., Quince, C., and Knight, R. (2011). UCHIME improves sensitivity and speed of chimera detection. *Bioinformatics* 27(16)**,** 2194-2200. doi: 10.1093/bioinformatics/btr381.

Magoč, T., and Salzberg, S.L. (2011). FLASH: fast length adjustment of short reads to improve genome assemblies. *Bioinformatics* 27(21)**,** 2957-2963. doi: 10.1093/bioinformatics/btr507.

Segata, N., Izard, J., Waldron, L., Gevers, D., Miropolsky, L., Garrett, W.S., et al. (2011). Metagenomic biomarker discovery and explanation. *Genome Biol* 12(6)**,** R60. doi: 10.1186/gb-2011-12-6-r60.
